# Quantitative or qualitative transcriptional diagnostic signatures? A case study for colorectal cancer

**DOI:** 10.1186/s12864-018-4446-y

**Published:** 2018-01-29

**Authors:** Qingzhou Guan, Haidan Yan, Yanhua Chen, Baotong Zheng, Hao Cai, Jun He, Kai Song, You Guo, Lu Ao, Huaping Liu, Wenyuan Zhao, Xianlong Wang, Zheng Guo

**Affiliations:** 1 Fujian Key Laboratory of Medical Bioinformatics, Key Laboratory of Ministry of Education for Gastrointestinal Cancer, Fujian Medical University, Fuzhou, 350122 China; 2 Fujian Key Laboratory of Tumor Microbiology, Fujian Medical University, Fuzhou, 350122 China; 3 College of Bioinformatics Science and Technology, Harbin Medical University, Harbin, 150086 China; 4 Department of Preventive Medicine, School of Basic Medicine Sciences, Gannan Medical University, Ganzhou, 341000 China

**Keywords:** Classifiers, Diagnostic signature, Relative expression orderings, Platform, Batch effects

## Abstract

**Background:**

Due to experimental batch effects, the application of a quantitative transcriptional signature for disease diagnoses commonly requires inter-sample data normalization, which would be hardly applicable under common clinical settings. Many cancers might have qualitative differences with the non-cancer states in the gene expression pattern. Therefore, it is reasonable to explore the power of qualitative diagnostic signatures which are robust against experimental batch effects and other random factors.

**Results:**

Firstly, using data of technical replicate samples from the MicroArray Quality Control (MAQC) project, we demonstrated that the low-throughput PCR-based technologies also exist large measurement variations for gene expression even when the samples were measured in the same test site. Then, we demonstrated the critical limitation of low stability for classifiers based on quantitative transcriptional signatures in applications to individual samples through a case study using a support vector machine and a naïve Bayesian classifier to discriminate colorectal cancer tissues from normal tissues. To address this problem, we identified a signature consisting of three gene pairs for discriminating colorectal cancer tissues from non-cancer (normal and inflammatory bowel disease) tissues based on within-sample relative expression orderings (REOs) of these gene pairs. The signature was well verified using 22 independent datasets measured by different microarray and RNA_seq platforms, obviating the need of inter-sample data normalization.

**Conclusions:**

Subtle quantitative information of gene expression measurements tends to be unstable under current technical conditions, which will introduce uncertainty to clinical applications of the quantitative transcriptional diagnostic signatures. For diagnosis of disease states with qualitative transcriptional characteristics, the qualitative REO-based signatures could be robustly applied to individual samples measured by different platforms.

**Electronic supplementary material:**

The online version of this article (10.1186/s12864-018-4446-y) contains supplementary material, which is available to authorized users.

## Background

In clinical, biopsy sampling with less-invasive techniques such as colonoscopy and endoscopic ultrasound-guided fine needle aspiration is often used for the initial clinical evaluation of cancer [1–6]. However, an indeterminate diagnosis often creates a dilemma [7]. Taking colorectal cancer as an example, it has been reported that the miss rate of colorectal cancer after colonoscopy, which is the predominant screening and diagnostic test for colorectal cancer [2, 8, 9], is about 15% for patients with inflammatory bowel diseases (IBD) [2]. Thus, it is necessary to find a molecular biomarker as an auxiliary diagnostic method for colonoscopy.

With the wide application of high throughput gene expression profiling techniques, many classifiers based on quantitative transcriptional signatures for cancer subtyping [10–12] or early detection [13–17] have been developed. However, clinical applications of these transcriptional signatures are scarce due to technological, mathematical and translational barriers [18]. Besides factors such like tissue sampling [19] and sample preparation quality [20], a well-known factor is that gene expression data are often “noisy” and subject to lab and batch effects introduced by the differences in laboratory conditions and personnel [21–23]. As reported by the MicroArray Quality Control (MAQC) project [24], for the high-throughput microarray platforms, the median values of coefficient of variation (CV) of gene expression levels in replicate samples measured by the same platforms ranged from 5 to 15% within the same test sites and became 10 to 20% for replicate samples measured across different test sites. Similarly, as demonstrated in this study, the quantitative measurements of gene expression in replicate samples measured by the low-throughput PCR-based technologies, such as Standardized (Sta) RT-PCR™ Assays and TaqMan® Gene Expression Assays, also exist large variations even in the same test sites. The large variation of quantitative measurements will introduce uncertainty of such signatures in applications. Due to this problem, the application of classical classifiers based on quantitative transcriptional signatures requires data normalization. This means that the analysis of a single sample requires this sample to be normalized along with a set of samples measured together. This constraint makes the classifiers hardly applicable under common clinical settings. Especially for prognostic signatures, the risk score of a patient is dependent on the risk composition of the other samples adopted for normalization together, introducing substantial uncertainty for risk predication [25–27].

Notably, among the vast number of reported quantitative disease signatures, several signatures have been approved by the Food and Drug Administration (FDA). One of the FDA approved signatures is MammaPrint® for predicting the recurrence risk of early stage (I and II) breast cancer patients with lymph node negative and tumor size < 5.0 cm treated with surgical resection [28–30]. However, currently the tissue samples must be sent to one of the two Agendia laboratories (one in Amsterdam, The Netherlands, and the other in Irvine, CA) for measurement with strict quality control and data normalization, which greatly limits the wide application of the signature. Another FDA approved signature is AlloMap® [31] for identifying the probability of transplant rejection for heart transplant recipients, which also requires patients’ samples to be sent to a central laboratory (XDx reference laboratory, based in Brisbane, California) [31, 32]. The same problem exists in other transcriptional signatures incorporated into clinical recommendations and guidelines, such like the Oncotype DX genomic assay (Genomic Health, Inc. Redwood City, CA, USA) used for predicting recurrence risk of early stage breast cancer and in decision making with respect to systemic therapy [33]. Therefore, obviation of the impact of the batch effects and the need of inter-sample normalization is an urgent issue.

In contrast, it has been found that the within-sample relative expression orderings (REOs) of gene pairs, which is also called Relative Expression Analysis (RXA) [34], are robust against experimental batch effects and invariant to monotone data transformation [34, 35]. Besides, the within-sample REOs of gene pairs are robust against variations of the tumor epithelial cell proportions in tissues sampled from different sites of a tumor [19, 36], partial RNA degradation in the sample preparation process and during the storage stage [20] and amplification bias for minimum specimens even with about 15–25 cancer cells [37], which are also important factors leading to the failure of validation and clinical application of the quantitative transcriptional signatures. The robustness property of the within-sample REOs enables researchers to integrate multiple datasets produced by the same or similar platforms for selecting disease signatures and training classifiers [20, 38, 39], which makes it more likely to find robust signatures [25, 38, 40]. Based on this unique advantage, some REO-type classifiers, such as TSP [41], K-TSP [42] and other adjusted methods [26, 43] were proposed to identify signatures for discriminating cancer subtypes [18, 38, 39, 44–46]. Recently, we have reported several REO-based prognostic signatures for specific medical issues for various cancers such as non-small cell lung cancer [25, 47], colorectal cancer [48] and other cancers [49–51], which have been well verified in multiple data sources produced by different laboratories, obviating the need of inter-sample data normalization. These results provide strong evidences of the clinical applicability of the type of signatures based on the robust qualitative REO information extracted from the quantitative measurements of gene expression, rather than the “exact” quantitative measurements themselves [52]. As revealed recently, although different platforms (e.g., Affymetrix and Illumina platforms) have different measurement principles, it would be highly likely that a REO-based signature consistently detected by two or more platforms could be robustly applied to samples measured by other platforms [53].

In this article, in addition to the previous results for the high-throughput platforms reported by the MicroArray Quality Control (MAQC) project [24], we firstly demonstrated that the quantitative values of gene expression in replicate samples measured by two low-throughput PCR-based technologies (StaRT-PCR™ Assays and TaqMan® Gene Expression Assays) in the same test site also exist large variations. Then, through a case study of building a support vector machine (SVM) and a naïve Bayesian classifier for discriminating colorectal cancer samples from normal samples, we demonstrated that the classical classifiers based on quantitative transcriptional signatures cannot be robustly applied to independent samples measured by the same platform used for the training data, let alone the samples measured by different platforms, which makes this type of signatures being hardly applicable under clinical settings. Then, we developed a within-sample REO-based signature that could discriminate colorectal cancer from non-cancer samples (IBD and normal samples) without the need of inter-sample data normalization or experimental batch adjustment. The signature was validated using data from multiple sources measured by different laboratories with different platforms.

## Results

### Technical variations of quantitative measurement

Firstly, we evaluated the CV of gene expression measurements in replicates for sample A and sample B measured in the same test site by two PCR-based technologies, StaRT-PCR™ Assays and TaqMan® Assays, respectively.

**Fig. 1:**
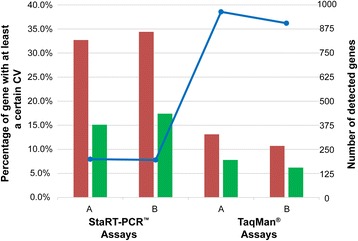
Quantitative measurement variation for replicates measured by PCR-based technologies. For each of the sample types (sample A and sample B) measured by StaRT-PCR™ Assays and TaqMan® Assays, the red bar denotes the percentage of genes that shows at least 10% CV and the green bar denotes the percentage of genes that shows at least 15% CV. The total number of such genes within each assay and sample type is noted by blue dots connected by lines and is read on the secondary axis

For a total of 199 genes with non-zero measurements assayed by StaRT-PCR™ for 3 replicates of sample A, about 32.7% genes showed at least 10% CV and 15.1% genes showed at least 15% CV. Similarly, for a total of 195 genes with non-zero measurements assayed by StaRT-PCR™ for 3 replicates of sample B, about 34.4% genes showed at least 10% CV and 17.4% genes showed at least 15% CV, the results were also shown in Fig. 1.

For a total of 964 genes with non-zero measurements assayed by TaqMan® for sample A, about 13.1% genes showed at least 10% CV and 7.8% genes showed at least 15% CV. Similarly, for a total of 905 genes with non-zero measurements assayed by TaqMan® for sample B, about 10.7% genes showed at least 10% CV and 6.2% genes showed at least 15% CV, as shown in Fig. 1. Although TaqMan® Assays showed smaller variations than StaRT-PCR™ Assays, the variations were still not negligible even in samples measured in the same test site, and it could expect that the variations would increase for measurements from different test sites.

### Limitation of classifiers based on quantitative transcriptional signatures

Due to large experimental batch effects, quantitative transcriptional measurement data from different experiments or profiled with different platforms could not be directly put together to train traditional SVM and naïve Bayesian classifiers. Because we could not find a single dataset with sufficient samples for colorectal cancer, normal and IBD tissues simultaneously, we were unable to train SVM and naïve Bayesian classifiers based on quantitative measurements for discriminating colorectal cancer and non-cancer (normal or IBD) tissue samples. Thus, we constructed the SVM and naïve Bayesian classifiers for a simpler problem, discriminating colorectal cancer and normal tissue samples, to demonstrate the limitations of quantitative transcriptional signatures.

**Fig. 2:**
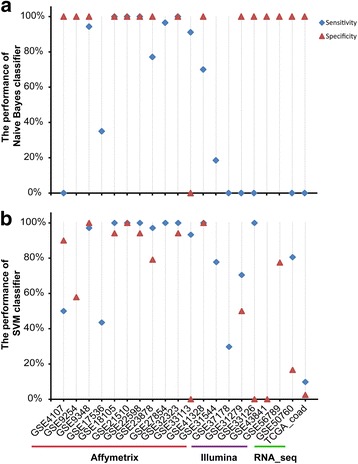
Sensitivity and specificity of SVM classifiers (**a**) and naïve Bayesian classifier (**b**) for validation datasets. Notably, some datasets included only colorectal cancer tissue samples or normal tissue samples, so only the results of sensitivity or specificity were shown for those datasets

Between the 32 cancer samples and 32 normal samples from dataset GSE8671, 7028 differentially expressed genes were detected using Student’s *t*-test with 1% FDR control. Using these 7028 genes as feature genes, a SVM classifier with radial basis function (RBF) kernel was trained with tenfold cross-validation [54, 55] using the training dataset GSE20916 with 91 cancer and 44 normal tissue samples. The sensitivity and specificity of the SVM classifier were 98.9% and 100.0% in the training dataset, respectively. However, when tested by validation datasets without applying inter-sample normalization, the classifier failed badly in many cases as described in Fig. 2a and Additional file 1: Table S1. For example, only 35.0% of the 177 cancer samples from the dataset GSE17536 were correctly classified and none of the 12 cancer samples from the dataset GSE4107 were correctly classified. Both the datasets were measured by the same Affymetrix platform with the training dataset. When the SVM classifier was applied to the datasets measured by other platforms, none of the 365 cancer samples from three datasets (GSE31279 measured by the Illumina platform; GSE50760 and TCGA measured by the RNA_seq platform) were correctly classified. Similar results were also observed for the naïve Bayesian classifier, as shown in Fig. 2b and Additional file 1: Table S1.

More comprehensive evaluation results were shown in Supplementary Result and Additional file 1: Table S2-S6. These results clearly show that the classical classifiers based on the quantitative transcriptional signatures cannot be robustly applied to independent samples even measured by the same platform as the training datasets, let alone the samples measured by different platforms. This problem limits the applicability of these classifiers to clinical applications.

### Identification and application of REO-based signature

**Fig. 3:**
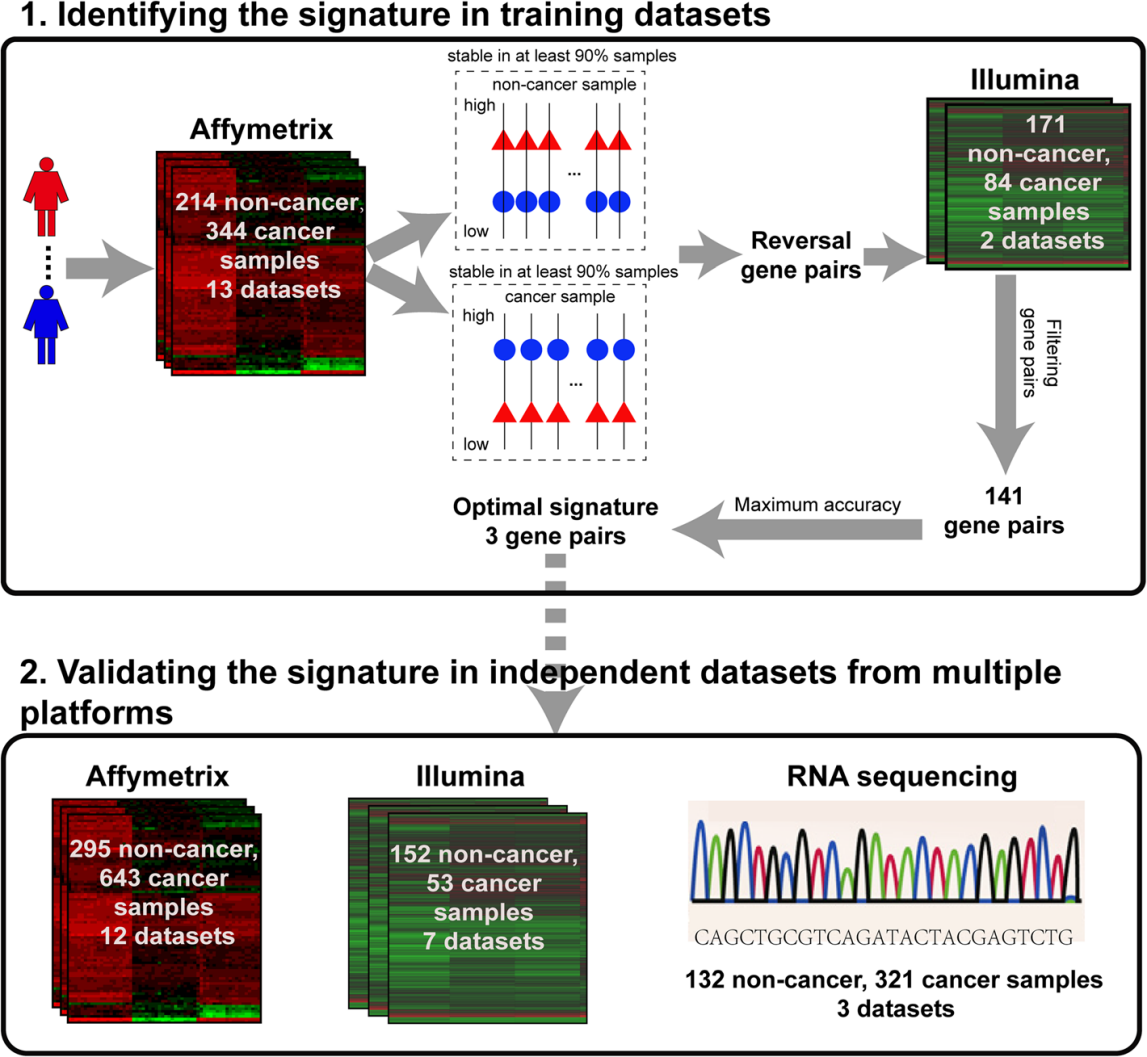
Analysis procedure for identifying a cross-platform REO-based signature

**Fig. 4:**
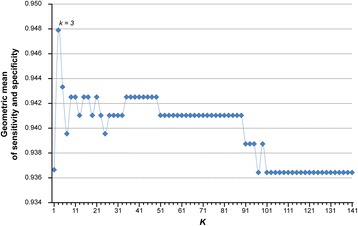
Performance of *k*-gene pairs REO-based signature applied to the training set. The majority vote rule was used for classification

The analysis procedure is described in Fig. 3. Firstly, using 91 normal samples and 123 IBD samples measured by the Affymetrix platform collected from 11 datasets (see Table 2), we identified 144,090,213 gene pairs with identical REOs in at least 90% of both the normal samples and the IBD samples. Similarly, using 344 colorectal cancer tissue samples from 9 datasets measured by the Affymetrix platform (see Table 2), we identified 149,446,895 gene pairs with identical REOs in at least 90% of the cancer tissues. We found 843 gene pairs that have reversal REOs from the above two lists of gene pairs. Among these 843 gene pairs, we further selected 141 gene pairs that had the identical REOs in at least 90% of 171 non-cancer samples and reversed REOs in at least 90% of 84 cancer samples in the combined GSE48634 and GSE37178 datasets measured by the Illumina platform, the list of the 141 gene pairs were shown in Additional file 1: Table S7. These 141 gene pairs were sorted in a descending order according to their reversal coverage rates (see Methods) between all the cancer samples and all the non-cancer samples in the training data collected from 13 datasets measured by Affymetrix platform (see Table 2). We then used the top-ranked *k* pairs, where *k* is an odd integer, to classify samples according to the majority vote rule. The results showed that for all possible *k* values ranging from 1 to 141, the largest geometric mean of sensitivity and specificity was 94.8% when *k* = 3 (Fig. 4). Thus, these three gene pairs, as described in Table 1, were selected as the signature for discriminating colorectal cancer samples from non-cancer samples.

**Table 1:** The REO-based signature

Gene pair	REO (*G*_*i*_ > *G*_*j*_)^a^
1	*GPAT3* > *TRIP13*
2	*PYY* > *CKAP2*
3	*SDCBP2* > *DAP3*

Note:

^a^Relative expression ordering (REO) of a gene pair, *G*_*i*_ > *G*_*j*_ denotes that the expression value of gene *i* is larger than the expression value of gene *j* in 90% of non-cancer samples but is less than the expression value of gene *j* in 90% of colorectal cancer samples

**Fig. 5:**
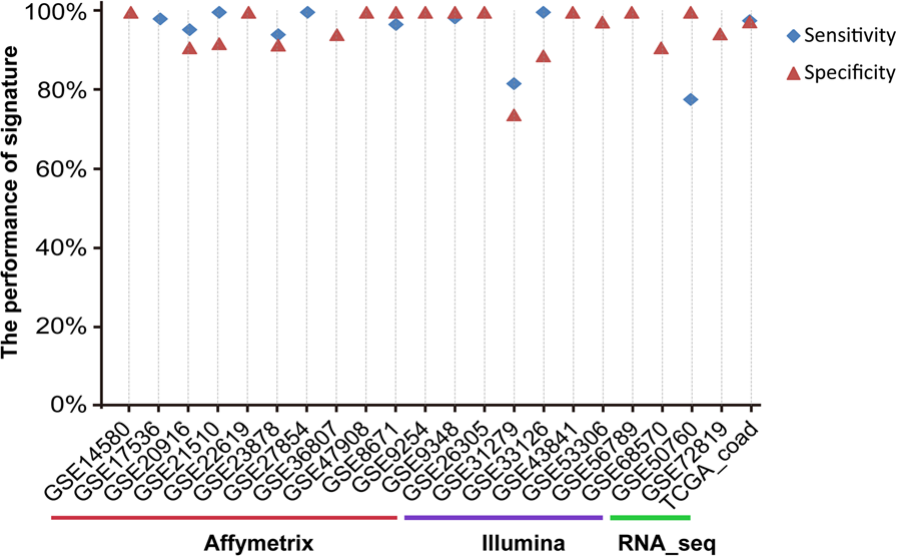
Performance of the REO-based signature applied to multiple independent datasets from different platforms. The majority vote rule was used for classification

The performance of the signature was evaluated using independent test datasets measured by multiple different platforms. As shown in Fig. 5 and Additional file 1: Table S8, the performance of the signature in each of the 12 datasets measured by the Affymetrix GPL570 platform is excellent. In total, 98.3% of the 643 colorectal cancer samples and 96.6% of the 295 non-cancer samples were identified correctly. Similar results were observed for the independent test datasets measured by the Illumina platforms, as shown in Fig. 5 and Additional file 1: Table S8. Especially, the signature was also verified in the datasets measured by the RNA sequencing platforms which have no data used in obtaining the signature. For the TCGA dataset, 97.9% of the 285 colorectal cancer samples and 97.6% of the 41 normal colorectal samples were identified correctly. For the GSE72819 dataset which did not include colorectal cancer samples, 94.5% of the 73 non-cancer tissue samples were correctly identified. The above results indicate that the classifier based on the within-sample REOs of gene pairs can be applied to the analysis of individual samples measured by different platforms, obviating the need of inter-sample data normalization.

Moreover, to explore the generalization of the signature, we used all the possible top-ranked *k* (where *k* is an odd integer) pairs from the 141 gene pairs to classify samples according to the majority vote rule. With different top-rank *k*, similar performances were achieved in the validation datasets, as shown in Additional file 2: Figure S1. However, for the dataset GSE68570, the classification performance decreased slightly when *k* increased to 77 or larger. The possible reason of the decreased performance for GSE68570 should be that the gene pairs with relatively low reversal rates in the training data might be unstable in data measured by other platforms [53]. In general, the generalization of the signature with three gene pairs is good enough.

## Discussion

We demonstrated that, besides high-throughput gene expression profiling platforms, the low-throughput PCR-based quantitative measurements also exist large variation in replicate samples measured in the same or different test sites. Thus, the classifiers based on quantitative transcriptional signatures could not be robustly applied to individual samples measured by the same platform as the training samples, let alone those individual samples measured by different platforms. This could explain the problem mentioned in Introduction that some quantitative transcriptional signatures approved by FDA or incorporated into clinical guidelines must be sent to a central laboratory for measurement with strict quality control and data normalization. Besides the batch effects, the quantitative measurements of gene expression are commonly affected by partial RNA degradation [20] and different sampling sites of tumor for the same patient [19], which will increase the uncertainty for clinical applications of quantitative transcriptional diagnostic signatures.

Fortunately, as demonstrated in our previous studies [19, 20, 36] and in this study, the REO-based transcriptional signatures could circumvent the above-mentioned problems. As a case study, we identified a signature consisting of three gene pairs for discriminating colorectal cancer from non-cancer (normal and IBD) tissue samples based on the within-sample REOs of the gene pairs. The result showed that the REO-based signature obtained from samples measured by two different platforms could be robustly applied to classify individual samples measured by multiple different platforms, including the RNA_seq platform that did not participate in the training process. However, in the GSE31279 dataset measured by the Illumina GPL6104 platform which did not participate in the training process, the signature performed relatively poor: only 81.8% of the 44 cancer samples and 73.8% of the 42 normal samples from were correctly identified. Although the within-sample REOs tend to be rather robust to data measured by different platforms, a certain degree of uncertainty still exists due to different measurement principles of the platforms [53]. Ideally, a REO-based signature should be applied to data measured by the platforms participating the train and validation of the signature.

**Fig. 6:**
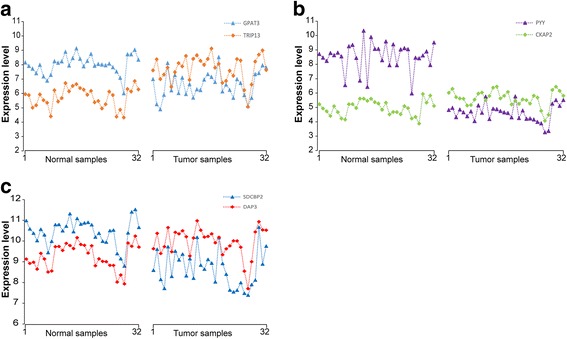
The distribution of the expression levels of the 3 gene-pairs in GSE8671. The gene expression levels of *GPAT3* and *TRIP13* (**a**), *PYY* and *CKAP2* (**b**) and *SDCBP2* and *DAP3* (**c**)

Even with sufficient high-quality data, it is difficult to interpret the signature used in complex classifiers to gain biological insights about the biomarkers [18]. In contrast, we can readily gain biological insights for a signature consisting of only a few genes. The three gene pairs of the signature for colorectal cancer diagnosis consist of *GPAT3* and *TRIP13*, *PYY* and *CKAP2*, *SDCBP2* and *DAP3*. These genes were found in the differentially expressed genes (Student’s *t*-test, FDR < 0.01) detected between the 32 cancer samples and 32 normal samples in the GSE8671 dataset. For *GPAT3*-*TRIP13* gene pair, both up-regulation of *TRIP13* and down-regulation of *GPAT3* contribute to the reversal REO in colorectal cancer samples. Similarly, for *PYY*-*CKAP2* and *SDCBP2*-*DAP3* gene pairs, up-regulation of *CKAP2*, *DAP3* and down-regulation of *PYY*, *SDCBP2* contribute to the reversal REO in colorectal cancer samples. Some of these genes, such as *TRIP13* [56], *PYY* [57], are known to be cancer-associated. *TRIP13* is a novel mitotic checkpoint-silencing protein, whose overexpression is associated with poor prognosis in breast cancer patients [56, 58, 59]. The decreased expression of *PYY* may be relevant to the development and progression of colon adenocarcinoma [57]. We additionally showed the distribution of the expression level of the 6 genes in dataset GSE8671. As shown in Fig. 6, the fold changes of each signature gene pair across samples for the two phenotypes were quite different. For the *GPAT3* - *TRIP13* gene pair, as shown in Fig. 6a, the fold change of the expression levels between *GPAT3* and *TRIP13* took values ranging from 1.26 to 1.72 with the median of 1.38 in the normal samples, while in the tumor samples the fold change took values ranging from 0.63 to 1.06 with the median of 0.87. Similar results for the other two gene pairs, *PYY* - *CKAP2* and *SDCBP2* - *DAP3*, were shown in Fig. 6b and c, respectively. The above results showed that the fold changes of each signature gene pair are quite different across different samples for each of the two phenotypes but the relative expression levels of the gene pair are stably.

The REO-based method is based on a single binary “switch” that compares the ordering of expression between two genes. The simplicity does not necessarily limit its prediction performance and the method is not prone to the overfitting issue. Arguably, REO-based signatures may lose some subtle quantitative information on gene expression. However, considering that subtle quantitative information of gene expression measurements tends to be unreliable and even the ratios of expression values of gene pairs are affected by the batch effects [25, 60], the apparent disadvantage of REOs analysis is in fact a unique advantage in terms of robustness [20]. The REO-based signature identified for colorectal cancer obviates the need of data normalization, which makes it feasible to clinical settings for colorectal cancer diagnosis and surveillance of patients with long-term IBD using biopsies obtained by colonoscopy or other improved techniques [61–65]. Notably, we have applied both the tissue samples and biopsy samples for training and validation. Thus, the signature based on the REOs is suitable for tissue samples and biopsy samples [10].

The main purpose of this study is to systematically demonstrate the critical limitations of the traditional classifiers based on the quantitative transcriptional measurements, which are sensitive to batch effects and detection platforms and could not be applied directly to the data measured by different laboratories. As for the REO-based method, other approaches, such as TSP and k-TSP, could be applied to the data measured by different laboratories or platforms. Here, we additionally evaluated other rank based approaches using the same training and validation datasets. Using the tspair R package (version 3.3.3), we trained the TSP classifier in the training samples directly combined from data measured by the Affymetrix and Illumina platforms. In the training set, 97.0% of the 428 cancer samples and 94.3% of the 385 non-cancer sample were correctly identified. However, the classifier failed badly in many validation datasets as described in Additional file 1: Table S9. Using the ktspair R package (version 3.3.3), we also trained the k-TSP classifier. In the training set, with the default fivefold cross-validation, 5 gene pairs were selected as the classification signature which correctly identified 96.7% of the 428 cancer samples and 98.0% of the 385 non-cancer sample. In the validation data, the k-TSP classifier performed better than the TSP classifier but poorer than our signature, as shown in Additional file 1: Table S10. For example, for the dataset GSE23878, our REO signature could identify 91.7% of the 24 non-caner sample correctly, but the k-TSP signature identified only 41.7% non-cancer samples correctly. One possible reason should be that the difference in the proportion of samples from Affymetrix and Illumina platform will make the signature to be unable to characterize the common features of the two platforms but biased to the platform with larger samples. Other approaches such as CART [66] should have the same problem. In the training process for our REO signature, the gene pairs (141 gene pairs) that were consistently detected in the data produced by the two platforms were used for the final signature selection (3 gene pairs in this study). Therefore, our method is intuitive and simple with the ability to identify very robust disease signatures.

In conclusion, REO-based signatures circumvent the critical limitation of quantitative transcriptional signatures and the REO-based classifying method should be also applicable for classifying other tissue samples. Moreover, because the data normalization problem also exists in miRNA [67] and DNA methylation profile analyses, the REO-based analysis of these multi-omic data should be taken into account in the further study.

## Conclusions

Because the subtle quantitative information of gene expression measurements currently tends to be greatly affected by many random factors, the disease diagnostic signatures based on the quantitative measurements lack robustness for clinical applications. Thus, we should make more efforts to capture the qualitative differences of gene expression patterns between cancer and non-cancer and between cancer subtypes to exploit robust qualitative signatures for disease diagnosis.

## Methods

### Data and preprocessing

The gene expression profiles analyzed in this study are described in Table 2. The array-based data measured by the Affymetrix and Illumina platforms were downloaded from Gene Expression Omnibus [68] (GEO, http://www.ncbi.nlm.nih.gov/geo/) and the mRNA-seq data measured by the Illumina platform were downloaded from ArrayExpress [69] (http://www.ebi.ac.uk/arrayexpress/) and The Cancer Genome Atlas [70] (TCGA, http://cancergenome.nih.gov/).

**Table 2:** Data used in this study

GEO Acc	Platform	Sample size^a^		
		Normal	IBD	Tumor
Training				
GSE32323	Affymetrix GPL570	17		17
GSE22598	Affymetrix GPL570	17		17
GSE41328	Affymetrix GPL570	10		10
GSE4107	Affymetrix GPL570	10		12
GSE4183	Affymetrix GPL570	8	15	15
GSE18105	Affymetrix GPL570	17		94
GSE12251	Affymetrix GPL570		23	
GSE13367	Affymetrix GPL570		16	
GSE9452	Affymetrix GPL570		8	
GSE16879	Affymetrix GPL570	6	61	
GSE35144	Affymetrix GPL570			27
GSE35896	Affymetrix GPL570			62
GSE33113	Affymetrix GPL570	6		90
GSE37178	Illumina GPL6947			84
GSE48634	Illumina GPL10558	69	102	
Validation				
GSE9348	Affymetrix GPL570	12		70
GSE23878	Affymetrix GPL570	24		35
GSE47908	Affymetrix GPL570	15	39	
GSE36807	Affymetrix GPL570	7	28	
GSE27854	Affymetrix GPL570			115
GSE22619	Affymetrix GPL570	10	10	
GSE21510	Affymetrix GPL570	25		123
GSE17536	Affymetrix GPL570			177
GSE14580	Affymetrix GPL570	6	24	
GSE8671	Affymetrix GPL570	32		32
GSE9254	Affymetrix GPL570	19		
GSE20916	Affymetrix GPL570	44		91
GSE53306	Illumina GPL10558	12	28	
GSE31279	Illumina GPL6104	42		44
GSE33126	Illumina GPL6947	9		9
GSE68570	Illumina GPL10558	5	6	
GSE26305	Illumina GPL6884	2	2	
GSE56789	Illumina GPL10558	40		
GSE43841	Illumina GPL14951	6		
GSE50760^b^	Illumina GPL11154	18		36
GSE72819^b^	Illumina GPL11154		73	
TCGA_coad^b,c^	IlluminaHiSeq_RNASeqV2	41		285

Notes:

^a^Empty cells indicate that there is no sample in the corresponding category

^b^These samples are measured by the RNA-sequencing platform

^c^Denotes the colorectal adenocarcinoma sample from TCGA

For the data measured by the Affymetrix platform, we downloaded the raw mRNA expression data (.CEL files) and used the Robust Multi-array Average (RMA) algorithm for background adjustment without quantile normalization [71]. For the data measured by the Illumina platform, we directly downloaded the processed data. For the sequence-based data from TCGA, we directly downloaded the level 3 data measured by the UNC IlluminaHiSeq_RNASeqV2 platform.

For the array-based data, each probe ID was mapped to Entrez gene ID with the corresponding platform file. If a probe was mapped to multiple or zero genes, then the data of this probe were deleted. If multiple probes were mapped to the same gene, the expression value of the gene was defined as the arithmetic mean of the values of multiple probes. For the sequence-based data from ArrayExpress, the gene symbols were mapped to Entrez gene ID with the biological database network [72] (bioDBnet, https://biodbnet-abcc.ncifcrf.gov/db/db2db.php).

### Variation analysis of quantitative measurement

In the MicroArray Quality Control (MAQC) project, two commercially available Reference RNA samples (sample A and sample B) with multiple replicates were measured by multiple microarray platforms and PCR-based technologies [24]. The MAQC project has reported the large measurement variations of the high-throughput microarray platforms [24]. Here, we additionally analyzed the variations of quantitative gene expression levels measured by two PCR-based technologies, Standardized (Sta) StaRT-PCR™ and TaqMan® Gene Expression Assays.

The MAQC PCR-based data, as described in Table 3, were directly downloaded from GSE5350. Notably, for the 3 replicates of sample A and sample B measured by StaRT-PCR™ Assays. If the measurement of a gene was 0 or “nan” in at least one replicate of a sample, then this gene was not included for further analysis. Thus, the total number of genes was not identical for sample A and sample B. For 4 replicates of sample A or sample B measured by TaqMan® Assay, a gene was considered absent in a sample when the average cycle threshold (CT) exceeds 35 [24]. For sample A or sample B, if the measurement of a gene was absent in at least one replicate, this gene was not included for the further analysis. Thus, the total number of genes for sample A and sample B obtained from TaqMan® Assays was also not identical.

**Table 3:** MAQC PCR-based data used in this study

GEO Acc	Protocol	Platform	Sample A	Sample B
GSE5350	StaRT-PCR™ Assays	GPL4198	3	3
GSE5350	TaqMan® Assays	GPL4097	4	4

For the gene expression levels of a certain gene in the replicates for sample A or sample B measured by each platform, the coefficient of variation (CV), calculated as the ratio of the standard deviation and arithmetic mean for the expression levels of this gene in the replicates, is used to measure the degree of variation of quantitative measurements. For the sample A and sample B measured by each platform, we calculated the percentage of genes that shows at least 10% and 15% CV, respectively, to reveal the degree of variation or uncertainty of quantitative measurements.

### SVM and naïve Bayesian classifiers

The SVM classifier using radial basis function (RBF) kernel [55] and the naïve Bayesian classifier, implanted in the WEKA software (version 3–6-13) with the default settings [54], were used for the case study. Each of the classifiers was trained with tenfold cross-validation in the training data. The performance of a trained classifier was evaluated in multiple independent data with or without normalization.

We called cancer samples as positive samples, non-cancer samples, either normal or IBD, as negative samples, and evaluated the performance of the classification signature using sensitivity and specificity which are calculated as follows:

$$ \mathrm{S}\mathrm{ensitivity}=\frac{\mathrm{TP}}{\mathrm{TP}+\mathrm{FN}} $$

$$ \mathrm{Specificity}=\frac{\mathrm{TN}}{\mathrm{TN}+\mathrm{FP}} $$

where TP, TN, FP and FN denote the number of true positives, true negatives, false positives and false negatives, respectively.

### Identification of the REO-based diagnosis signature

First, in the training dataset, each gene measurement is converted to its rank within each sample (the smallest measurement corresponding to the minimum rank, and the largest measurement corresponding to the maximum rank). Then, pairwise comparisons are performed for all genes to identify gene pairs with stable ordering in samples for a particular tissue type. For a pair of genes (*i*, *j*), the relationship of their relative ranks, G_*i*_ and G_*j*_, within one sample, has only two possibilities, G_*i*_ > G_*j*_ or G_*i*_ < G_*j*_. The relationship is called the relative expression ordering (REO). If the same REO pattern is maintained in a majority of samples, e.g. 90%, it is called a highly stable REO and the pair is a highly stable gene pair. Furthermore, if a gene pair (*i*, *j*) is highly stable in both a group of non-cancer samples and a group of cancer samples, respectively, but with reversal REO patterns (G_*i*_ < G_*j*_ in one group but G_*i*_ > G_*j*_ in the other group), the pair is called a reversal gene pair. Here, we selected the reversal gene pairs which are highly stable in non-cancer samples and cancer samples, respectively, but the REO patterns are reversed in the latter group. They form the candidate REO signature of the cancer.

Then, the candidate REO signatures selected above were sorted in a descending order according to their reversal coverage rates, where the reversal coverage rate of a reversal gene pair is defined as the geometric mean of the percentage of the highly stable REO pattern in the non-cancer samples and the percentage of the reversed REO pattern in the cancer samples. Obviously, the higher the reversal coverage rate is for a gene pair, the higher the classification ability is for this gene pair.

Thirdly we used the top *k* gene pairs, where *k* is an odd integer ranging from 1 to the total number of the reversal gene pairs, to classify the samples based on the majority vote rule. The value of *k* was chosen as the smallest number of gene pairs that reached the highest geometric mean of the sensitivity and specificity in the training data.

Finally, the signature was tested in independent samples.

## Additional files

Additional file 1: Supplementary results for this manuscript. (DOCX 57 kb)

Additional file 2: Figure S1. Performances of all possible top-ranked *k* (from 1 to 141, *k* is an odd integer) gene pairs in the independent datasets measured by different platforms. The majority vote rule was used for classification. (TIFF 1848 kb)

## Abbreviations

CT: Cycle threshold
CV: Coefficient of variation
GEO: Gene Expression Omnibus
IBD: Inflammatory bowel diseases
MAQC: MicroArray Quality Control
RBF: Radial basis function
REO: Relative expression ordering
RMA: Robust Multi-array Average
RXA: Relative Expression Analysis
SVM: Support vector machine
TCGA: The Cancer Genome Atlas
