# Supplementary material for: Quantitative or qualitative transcriptional diagnostic signatures? A case study for colorectal cancer
Source: BMC Genomics. 2018 Jan 29;19:99. doi: 10.1186/s12864-018-4446-y (PMC5789529; doi:10.1186/s12864-018-4446-y)
Supplement: Supplementary file 1 — Supplementary results for this manuscript. (DOCX 57 kb) [file 12864_2018_4446_MOESM1_ESM.docx]

**Supplementary Result**

**Evaluation of the performance of SVM and naïve Bayesian classifiers**

Here, to make the comparison more fairly, the same datasets used in the REO approach were used to train and validate the performance of the SVM and naïve Bayesian classifiers. Between the 15 cancer samples and 23 non-cancer (normal and IBD) samples from the training dataset GSE4183, 158 differentially expressed genes (DEGs) were detected using Student's t-test with 1% FDR control. Using these 158 genes as feature genes and the remained training set with 413 cancer samples and 362 non-cancer tissue samples, a SVM classifier with radial basis function (RBF) kernel was trained with tenfold cross-validation. When the data were directly merged, the sensitivity and specificity of the SVM classifier were 98.3% and 97.5%, respectively, in the training dataset. However, when tested in the validation datasets, the classifier failed badly in many cases as described in Supplementary Table S2. Similar results were also observed for naïve Bayesian classifier, as described in Supplementary Table S2. In addition, because the REO-based signature contains 6 genes, we also selected the top 6 most significant DEGs identified from training set as the feature genes to construct SVM and naïve Bayesian classifiers. Similarly, when tested in the validation datasets, the classifier failed badly in many cases as described in Supplementary Table S3.

In the above analysis process, we directly merge the dataset from different experiments to construct the traditional classification model (SVM and naïve Bayesian) without considering the batch effects. Then, all the validation datasets were processed to remove batch effects with respect to the training set using the ComBat function [PMID:16632515] in the sva R package (version3 .3.3). However, no matter the all or the top 6 most significant DEGs identified from dataset GSE4183 were selected as feature genes, both the SVM and naïve Bayesian classifiers even could not be well trained in the training data. For example, based on all DEGs from GSE4183 as feature gene, the sensitivity and specificity of the SVM model in the training set was 100.0% and 8.8%, respectively. For naïve Bayesian model, the sensitivity and specificity in the training set was 78.5% and 43.1%, respectively. When the top 6 most significant DEGs from GSE4183 were selected as feature gene, similar results were obtained (data not shown).

We additionally used the DEGs from the dataset GSE22598, GSE32323 and GSE23878 as the feature genes, respectively, to construct the SVM and naïve Bayesian classifiers. Similarly, unstable results were also obtained in validation datasets, as shown in Supplementary Table S4-S6.

The above results further demonstrated that the limitation of classifiers based on quantitative transcriptional signatures.

**Supplementary Table**

**Table S1.** **Performance of the SVM and naïve Bayesian classifiers based on the quantitative transcriptional signatures**

| GEO Acc | Sample size* | | SVM^#^ | | Bayesian^#^ | |
| --- | --- | --- | --- | --- | --- | --- |
|  | Normal | Tumor | Sensitivity | Specificity | Sensitivity | Specificity |
| **Microarray Affymetrix** | | | | | | |
| GSE4107 | 10 | 12 | 0.0% | 100.0% | 50.0% | 90.0% |
| GSE9254 | 19 |  |  | 100.0% |  | 57.9% |
| GSE9348 | 12 | 70 | 94.3% | 100.0% | 97.1% | 100.0% |
| GSE17536 |  | 177 | 35.0% |  | 43.5% |  |
| GSE18105 | 17 | 94 | 100.0% | 100.0% | 100.0% | 94.1% |
| GSE21510 | 25 | 123 | 100.0% | 100.0% | 100.0% | 100.0% |
| GSE22598 | 17 | 17 | 100.0% | 100.0% | 100.0% | 94.1% |
| GSE23878 | 24 | 35 | 77.1% | 100.0% | 97.1% | 79.2% |
| GSE27854 |  | 115 | 96.5% |  | 100.0% |  |
| GSE32323 | 17 | 17 | 100.0% | 100.0% | 100.0% | 94.1% |
| GSE33113 | 6 | 90 | 91.1% | 0.0% | 93.3% | 0.0% |
| GSE41328 | 10 | 10 | 70.0% | 100.0% | 100.0% | 100.0% |
| GSE35144 |  | 27 | 18.5% |  | 77.8% |  |
| **Microarray Illumina** | | | | | | |
| GSE37178 |  | 84 | 0.0% |  | 29.8% |  |
| GSE31279 | 42 | 44 | 0.0% | 100.0% | 70.5% | 50.0% |
| GSE33126 | 9 | 9 | 0.0% | 100.0% | 100.0% | 0.0% |
| GSE43841 | 6 |  |  | 100.0% |  | 0.0% |
| GSE56789 | 40 |  |  | 100.0% |  | 77.5% |
| **RNA_seq** | | | | | | |
| GSE50760 | 18 | 36 | 0.0% | 100.0% | 80.6% | 16.7% |
| TCGA_coad^†^ | 41 | 285 | 0.0% | 100.0% | 9.8% | 2.4% |

Note: *Empty cells indicate that there is no sample in the corresponding category;

^#^Empty cells indicate that there is no corresponding result because no the corresponding data;

^†^The colorectal adenocarcinoma samples from TCGA.

**Table S2. Performances of the SVM and naïve Bayesian classifiers based on all differentially expressed genes from GSE4183**

| GEO Acc | Sample size* | | SVM^#^ | | Bayesian^#^ | |
| --- | --- | --- | --- | --- | --- | --- |
|  | Non-tumor | Tumor | Sensitivity | Specificity | Sensitivity | Specificity |
| **Microarray Affymetrix** | | | | | | |
| GSE8671 | 32 | 32 | 68.8% | 100.0% | 9.4% | 100.0% |
| GSE9254 | 19 |  |  | 10.5% |  | 15.8% |
| GSE9348 | 12 | 70 | 97.1% | 100.0% | 5.7% | 100.0% |
| GSE14580 | 30 |  |  | 100.0% |  | 100.0% |
| GSE17536 |  | 177 | 96.1% |  | 92.1% |  |
| GSE20916 | 44 | 91 | 92.3% | 72.7% | 12.1% | 54.6% |
| GSE21510 | 25 | 123 | 100.0% | 100.0% | 92.70% | 92.0% |
| GSE22619 | 20 |  |  | 70.0% |  | 75.0% |
| GSE23878 | 24 | 35 | 88.7% | 75.0% | 8.6% | 100.0% |
| GSE27854 |  | 115 | 98.3% |  | 93.0% |  |
| GSE36807 | 35 |  |  | 100.0% |  | 100.0% |
| GSE47908 | 54 |  |  | 94.4% |  | 64.8% |
| **Microarray Illumina** | | | | | | |
| GSE26305 | 4 |  |  | 0.0% |  | 0.0% |
| GSE31279 | 42 | 44 | 13.6% | 88.1% | 18.2% | 97.6% |
| GSE33126 | 9 | 9 | 100.0% |  | 100.0% | 0.0% |
| GSE43841 | 6 |  |  | 0.0% |  | 0.0% |
| GSE53306 | 40 |  |  | 0.0% |  | 0.0% |
| GSE56789 | 40 |  |  | 100.0% |  | 100.0% |
| GSE68570 | 11 |  |  | 18.2% |  | 100.0% |
| RNA_seq | | | | | | |
| GSE50760 | 18 | 36 | 100.0% | 0.0% | 100.0% | 0.0% |
| GSE72819 | 73 |  |  | 0.0% |  | 0.0% |
| TCGA_coad^†^ | 41 | 285 | 100.0% | 0.0% | 94.0% | 0.0% |

Note: *Empty cells indicate that there is no sample in the corresponding category;

^#^Empty cells indicate that there is no corresponding result because no the corresponding data;

^†^The colorectal adenocarcinoma samples from TCGA.

**Table S3. Performances of the SVM and naïve Bayesian classifiers based on the top 6 most significant DEGs identified from GSE4183**

| GEO Acc | Sample size* | | SVM^#^ | | Bayesian^#^ | |
| --- | --- | --- | --- | --- | --- | --- |
|  | Non-tumor | Tumor | Sensitivity | Specificity | Sensitivity | Specificity |
| **Microarray Affymetrix** | | | | | | |
| GSE8671 | 32 | 32 | 90.6% | 84.4% | 68.8% | 90.6% |
| GSE9254 | 19 |  |  | 5.3% |  | 5.3% |
| GSE9348 | 12 | 70 | 88.6% | 83.3% | 62.9% | 100.0% |
| GSE14580 | 30 |  |  | 96.7% |  | 100.0% |
| GSE17536 |  | 177 | 93.2% |  | 95.5% |  |
| GSE20916 | 44 | 91 | 73.6% | 43.2% | 36.3% | 54.6% |
| GSE21510 | 25 | 123 | 100.0% | 64.0% | 100.0% | 52.0% |
| GSE22619 | 20 |  |  | 40.0% |  | 80.0% |
| GSE23878 | 24 | 35 | 48.6% | 66.7% | 40.0% | 62.5% |
| GSE27854 |  | 115 | 98.3% |  | 98.3% |  |
| GSE36807 | 35 |  |  | 88.6% |  | 94.3% |
| GSE47908 | 54 |  |  | 37.0% |  | 48.2% |
| **Microarray Illumina** | | | | | | |
| GSE26305 | 4 |  |  | 0.0% |  | 25.0% |
| GSE31279 | 42 | 44 | 2.3% | 85.2% | 0.0% | 100.0% |
| GSE33126 | 9 | 9 | 100.0% | 0.0% | 100.0% | 0.0% |
| GSE43841 | 6 |  |  | 0.0% |  | 0.0% |
| GSE53306 | 40 |  |  | 0.0% |  | 0.0% |
| GSE56789 | 40 |  |  | 100.0% |  | 100.0% |
| GSE68570 | 11 |  |  | 63.6% |  | 100.0% |
| **RNA_seq** | | | | | | |
| GSE50760 | 18 | 36 | 100.0% | 0.0% | 94.5% | 11.1% |
| GSE72819 | 73 |  |  | 0.0% |  | 4.1% |
| TCGA_coad^†^ | 41 | 285 | 100.0% | 0.0% | 100.0% | 0.0% |

Note: *Empty cells indicate that there is no sample in the corresponding category;

^#^Empty cells indicate that there is no corresponding result because no the corresponding data;

^†^The colorectal adenocarcinoma samples from TCGA.

**Table S4. Performances of the SVM and naïve Bayesian classifiers based on the feature gene from dataset GSE22598**

| GEO Acc | Sample size* | | SVM^#^ | | Bayesian^#^ | |
| --- | --- | --- | --- | --- | --- | --- |
|  | Normal | Tumor | Sensitivity | Specificity | Sensitivity | Specificity |
| **Microarray Affymetrix** | | | | | | |
| GSE4107 | 10 | 12 | 0.0% | 100.0% | 83.3% | 30.0% |
| GSE8671 | 32 | 32 | 90.9% | 100.0% | 100.0% | 100.0% |
| GSE9254 | 19 |  |  | 100.0% |  | 10.5% |
| GSE9348 | 12 | 70 | 94.3% | 100.0% | 100.0% | 100.0% |
| GSE17536 |  | 177 | 33.9% |  | 48.0% |  |
| GSE18105 | 17 | 94 | 100.0% | 100.0% | 100.0% | 88.2% |
| GSE21510 | 25 | 123 | 100.0% | 100.0% | 100.0% | 96.0% |
| GSE23878 | 24 | 35 | 77.1% | 100.0% | 100.0% | 75.0% |
| GSE27854 |  | 115 | 96.5% |  | 100.0% |  |
| GSE32323 | 17 | 17 | 100.0% | 100.0% | 100.0% | 94.1% |
| GSE33113 | 6 | 90 | 91.1% | 0.0% | 93.3% | 0.0% |
| GSE41328 | 10 | 10 | 70.0% | 100.0% | 100.0% | 100.0% |
| GSE35144 |  | 27 | 14.8% |  | 63.0% |  |
| **Microarray Illumina** | | | | | | |
| GSE37178 |  | 84 | 0.0% |  | 60.7% |  |
| GSE31279 | 42 | 44 | 0.0% | 100.0% | 86.4% | 50.0% |
| GSE33126 | 9 | 9 | 0.0% | 100.0% | 100.0% | 0.0% |
| GSE43841 | 6 |  |  | 100.0% |  | 0.0% |
| GSE56789 | 40 |  |  | 100.0% |  | 97.5% |
| **RNA_seq** | | | | | | |
| GSE50760 | 18 | 36 | 0.0% | 100.0% | 75.0% | 55.6% |
| TCGA_coad† | 41 | 285 | 0.0% | 100.0% | 14.7% | 2.4% |

Note: *Empty cells indicate that there is no sample in the corresponding category;

^#^Empty cells indicate that there is no corresponding result because no the corresponding data;

^†^The colorectal adenocarcinoma samples from TCGA.

**Table S5. Performances of the SVM and naïve Bayesian classifiers based on the feature gene from dataset GSE32323**

| GEO Acc | Sample size* | | SVM^#^ | | Bayesian^#^ | |
| --- | --- | --- | --- | --- | --- | --- |
|  | Normal | Tumor | Sensitivity | Specificity | Sensitivity | Specificity |
| **Microarray Affymetrix** | | | | | | |
| GSE4107 | 10 | 12 | 0.0% | 100.0% | 83.3% | 50.0% |
| GSE8671 | 32 | 32 | 93.8% | 100.0% | 100.0% | 100.0% |
| GSE9254 | 19 |  |  | 100.0% |  | 15.8% |
| GSE9348 | 12 | 70 | 94.3% | 100.0% | 100.0% | 100.0% |
| GSE17536 |  | 177 | 37.9% |  | 45.8% |  |
| GSE18105 | 17 | 94 | 100.0% | 100.0% | 100.0% | 88.2% |
| GSE21510 | 25 | 123 | 100.0% | 100.0% | 100.0% | 96.0% |
| GSE22598 | 17 | 17 | 100.0% | 100.0% | 100.0% | 94.1% |
| GSE23878 | 24 | 35 | 77.1% | 100.0% | 100.0% | 75.0% |
| GSE27854 |  | 115 | 96.5% |  | 100.0% |  |
| GSE33113 | 6 | 90 | 91.1% | 0.0% | 93.3% | 0.0% |
| GSE41328 | 10 | 10 | 70.0% | 100.0% | 100.0% | 100.0% |
| GSE35144 |  | 27 | 14.8% |  | 77.8% |  |
| **Microarray Illumina** | | | | | | |
| GSE37178 |  | 84 | 0.0% |  | 25.0% |  |
| GSE31279 | 42 | 44 | 0.0% | 100.0% | 77.3% | 57.1% |
| GSE33126 | 9 | 9 | 0.0% | 100.0% | 100.0% | 0.0% |
| GSE43841 | 6 |  |  | 100.0% |  | 0.0% |
| GSE56789 | 40 |  |  | 100.0% |  | 97.5% |
| **RNA_seq** | | | | | | |
| GSE50760 | 18 | 36 | 0.0% | 100.0% | 72.2% | 50.0% |
| TCGA_coad† | 41 | 285 | 0.0% | 100.0% | 17.2% | 2.4% |

Note: *Empty cells indicate that there is no sample in the corresponding category;

^#^Empty cells indicate that there is no corresponding result because no the corresponding data;

^†^The colorectal adenocarcinoma samples from TCGA.

**Table S6. Performances of the SVM and naïve Bayesian classifiers based on the feature gene from dataset GSE23878**

| GEO Acc | Sample size* | | SVM^#^ | | Bayesian^#^ | |
| --- | --- | --- | --- | --- | --- | --- |
|  | Normal | Tumor | Sensitivity | Specificity | Sensitivity | Specificity |
| **Microarray Affymetrix** | | | | | | |
| GSE4107 | 10 | 12 | 0.0% | 100.0% | 100.0% | 20.0% |
| GSE8671 | 32 | 32 | 90.6% | 100.0% | 100.0% | 93.8% |
| GSE9254 | 19 |  |  | 100.0% |  | 5.3% |
| GSE9348 | 12 | 70 | 94.3% | 100.0% | 100.0% | 100.0% |
| GSE17536 |  | 177 | 35.0% |  | 51.4% |  |
| GSE18105 | 17 | 94 | 100.0% | 100.0% | 100.0% | 70.6% |
| GSE21510 | 25 | 123 | 100.0% | 100.0% | 100.0% | 96.0% |
| GSE22598 | 17 | 17 | 100.0% | 100.0% | 100.0% | 76.5% |
| GSE27854 |  | 115 | 96.5% |  | 100.0% |  |
| GSE32323 | 17 | 17 | 100.0% | 100.0% | 100.0% | 82.4% |
| GSE33113 | 6 | 90 | 89.0% | 0.0% | 94.4% | 0.0% |
| GSE41328 | 10 | 10 | 70.0% | 100.0% | 100.0% | 90.0% |
| GSE35144 |  | 27 | 18.5% |  | 29.3% |  |
| **Microarray Illumina** | | | | | | |
| GSE37178 |  | 84 | 0.0% |  | 59.5% |  |
| GSE31279 | 42 | 44 | 0.0% | 100.0% | 72.7% | 57.1% |
| GSE33126 | 9 | 9 | 0.0% | 100.0% | 100.0% | 0.0% |
| GSE43841 | 6 |  |  | 100.0% |  | 0.0% |
| GSE56789 | 40 |  |  | 100.0% |  | 10.0% |
| **RNA_seq** | | | | | | |
| GSE50760 | 18 | 36 | 0.0% | 100.0% | 75.0% | 61.1% |
| TCGA_coad† | 41 | 285 | 0.0% | 100.0% | 10.9% | 2.4% |

Note: *Empty cells indicate that there is no sample in the corresponding category;

^#^Empty cells indicate that there is no corresponding result because no the corresponding data;

^†^The colorectal adenocarcinoma samples from TCGA.

**Table S7. The Entrez Gene ID list of the 141 gene pairs**

| Gene 1 | Gene 2 |
| --- | --- |
| 84803 | 9319 |
| 5697 | 26586 |
| 27111 | 7818 |
| 84803 | 54517 |
| 84803 | 157567 |
| 84803 | 22976 |
| 6689 | 84126 |
| 51150 | 1019 |
| 6689 | 60436 |
| 84803 | 23658 |
| 5697 | 221830 |
| 84803 | 57187 |
| 84803 | 152189 |
| 80028 | 2118 |
| 2875 | 4609 |
| 6689 | 54876 |
| 6689 | 29902 |
| 84830 | 8507 |
| 9540 | 29105 |
| 2683 | 57761 |
| 6689 | 116447 |
| 6689 | 54441 |
| 5697 | 11062 |
| 6689 | 26586 |
| 6689 | 4487 |
| 84803 | 9533 |
| 84803 | 10845 |
| 6689 | 1951 |
| 84803 | 8540 |
| 84803 | 8725 |
| 134285 | 29899 |
| 5794 | 221908 |
| 84803 | 5638 |
| 6689 | 221830 |
| 84803 | 119391 |
| 5794 | 2648 |
| 134285 | 55299 |
| 27111 | 26996 |
| 6689 | 79810 |
| 84803 | 10127 |
| 5794 | 9533 |
| 84803 | 55149 |
| 11148 | 430 |
| 27111 | 6558 |
| 84803 | 201965 |
| 84803 | 26225 |
| 27111 | 51650 |
| 84803 | 203427 |
| 6689 | 7625 |
| 6689 | 261734 |
| 6689 | 65094 |
| 5794 | 159090 |
| 6750 | 79070 |
| 123264 | 84133 |
| 84803 | 7465 |
| 6689 | 10695 |
| 84803 | 54802 |
| 84888 | 10576 |
| 6689 | 81848 |
| 6689 | 115098 |
| 84803 | 51805 |
| 7851 | 10134 |
| 3570 | 79612 |
| 29948 | 2118 |
| 5794 | 90809 |
| 5794 | 54934 |
| 199964 | 4331 |
| 84803 | 23172 |
| 6689 | 125111 |
| 5152 | 80746 |
| 6689 | 10495 |
| 6689 | 4798 |
| 84803 | 6259 |
| 84803 | 79791 |
| 5794 | 23534 |
| 146223 | 54517 |
| 6689 | 84168 |
| 6799 | 57820 |
| 6689 | 80019 |
| 79153 | 22916 |
| 10066 | 84886 |
| 9540 | 197370 |
| 6689 | 51105 |
| 2981 | 57761 |
| 23563 | 1503 |
| 5794 | 51259 |
| 79153 | 10127 |
| 5794 | 10127 |
| 84803 | 84319 |
| 84803 | 79833 |
| 5794 | 54663 |
| 5794 | 84246 |
| 27111 | 6385 |
| 23563 | 10491 |
| 6689 | 147929 |
| 84803 | 83879 |
| 84803 | 22929 |
| 79153 | 3149 |
| 57171 | 54517 |
| 199964 | 54955 |
| 54509 | 11072 |
| 1212 | 1434 |
| 6689 | 828 |
| 84803 | 1459 |
| 4648 | 4487 |
| 8671 | 80746 |
| 54751 | 22880 |
| 79817 | 51366 |
| 25771 | 22880 |
| 27111 | 6879 |
| 1908 | 64327 |
| 79153 | 9569 |
| 6689 | 54434 |
| 84803 | 89958 |
| 84803 | 9419 |
| 151195 | 22880 |
| 5794 | 84798 |
| 84803 | 26276 |
| 6689 | 5255 |
| 6689 | 9185 |
| 84803 | 148479 |
| 6689 | 54881 |
| 84803 | 374659 |
| 4084 | 8507 |
| 5697 | 55308 |
| 84803 | 54663 |
| 6689 | 58509 |
| 79153 | 6873 |
| 84830 | 94234 |
| 83547 | 9569 |
| 84803 | 51720 |
| 84803 | 26009 |
| 2185 | 11072 |
| 64922 | 54517 |
| 84803 | 8662 |
| 5794 | 84890 |
| 5794 | 9128 |
| 23417 | 22880 |
| 79153 | 5383 |
| 203260 | 22880 |
| 57016 | 191 |

**Table S8.** **Performance of the REO-based signature (*k* = 3)**

| GEO Acc | Sample size* | | Sensitivity^#^ | Specificity^#^ |
| --- | --- | --- | --- | --- |
|  | Non-cancer | Cancer |  |  |
| **Microarray Affymetrix** | | | | |
| GSE14580 | 30 |  |  | 100.0% |
| GSE17536 |  | 177 | 98.3% |  |
| GSE20916 | 44 | 91 | 95.6% | 90.9% |
| GSE21510 | 25 | 123 | 100.0% | 92.0% |
| GSE22619 | 20 |  |  | 100.0% |
| GSE23878 | 24 | 35 | 94.3% | 91.7% |
| GSE27854 |  | 115 | 100.0% |  |
| GSE36807 | 35 |  |  | 94.3% |
| GSE47908 | 54 |  |  | 100.0% |
| GSE8671 | 32 | 32 | 96.9% | 100.0% |
| GSE9254 | 19 |  |  | 100.0% |
| GSE9348 | 12 | 70 | 98.6% | 100.0% |
| **Microarray Illumina** | | | | |
| GSE26305 | 4 |  |  | 100.0% |
| GSE31279 | 42 | 44 | 81.8% | 73.8% |
| GSE33126 | 9 | 9 | 100.0% | 88.9% |
| GSE43841 | 6 |  |  | 100.0% |
| GSE53306 | 40 |  |  | 97.5% |
| GSE56789 | 40 |  |  | 100.0% |
| GSE68570 | 11 |  |  | 90.9% |
| **RNA_seq** | | | | |
| GSE50760 | 18 | 36 | 77.8% | 100.0% |
| GSE72819 | 73 |  |  | 94.5% |
| TCGA_coad† | 41 | 285 | 97.9% | 97.6% |

Note: *Empty cells indicate that there is no sample in the corresponding category;

^#^Empty cells indicate that there is no corresponding result because no the corresponding data;

^†^The colorectal adenocarcinoma samples from TCGA.

**Table S9. Performances of the TSP signature**

| GEO Acc | Sample size* | | Sensitivity^#^ | Specificity^#^ |
| --- | --- | --- | --- | --- |
|  | Non-cancer | Cancer |  |  |
| **Microarray Affymetrix** | | | | |
| GSE14580 | 30 |  |  | 100.0% |
| GSE17536 |  | 177 | 99.4% |  |
| GSE20916 | 44 | 91 | 87.9% | 93.2% |
| GSE21510 | 25 | 123 | 95.9% | 92.0% |
| GSE22619 | 20 |  |  | 70.0% |
| GSE23878 | 24 | 35 | 91.4% | 29.2% |
| GSE27854 |  | 115 | 95.7% |  |
| GSE36807 | 35 |  |  | 62.9% |
| GSE47908 | 54 |  |  | 87.0% |
| GSE8671 | 32 | 32 | 93.8% | 40.6% |
| GSE9254 | 19 |  |  | 68.4% |
| GSE9348 | 12 | 70 | 100.0% | 66.7% |
| **Microarray Illumina** | | | | |
| GSE26305 | 4 |  |  | 50.0% |
| GSE31279 | 42 | 44 | 90.9% | 40.5% |
| GSE33126 | 9 | 9 | 100.0% | 0.0% |
| GSE43841 | 6 |  |  | 100.0% |
| GSE53306 | 40 |  |  | 100.0% |
| GSE56789 | 40 |  |  | 62.5% |
| GSE68570 | 11 |  |  | 90.9% |
| **RNA_seq** | | | | |
| GSE50760 | 18 | 36 | 75.0% | 100.0% |
| GSE72819 | 73 |  |  | 100.0% |
| TCGA_coad† | 41 | 285 | 98.3% | 56.1% |

Note: *Empty cells indicate that there is no sample in the corresponding category;

^#^Empty cells indicate that there is no corresponding result because no the corresponding data;

^†^The colorectal adenocarcinoma samples from TCGA.

**Table S10. Performances of the k-TSP signature**

| GEO Acc | Sample size* | | Sensitivity^#^ | Specificity^#^ |
| --- | --- | --- | --- | --- |
|  | Non-cancer | Cancer |  |  |
| **Microarray Affymetrix** | | | | |
| GSE14580 | 30 |  |  | 100.0% |
| GSE17536 |  | 177 | 100.0% |  |
| GSE20916 | 44 | 91 | 89.0% | 90.9% |
| GSE21510 | 25 | 123 | 100.0% | 96.0% |
| GSE22619 | 20 |  |  | 100.0% |
| GSE23878 | 24 | 35 | 100.0% | 41.7% |
| GSE27854 |  | 115 | 100.0% |  |
| GSE36807 | 35 |  |  | 91.4% |
| GSE47908 | 54 |  |  | 98.1% |
| GSE8671 | 32 | 32 | 81.3% | 100.0% |
| GSE9254 | 19 |  |  | 78.9% |
| GSE9348 | 12 | 70 | 100.0% | 91.7% |
| **Microarray Illumina** | | | | |
| GSE26305 | 4 |  |  | 100.0% |
| GSE31279 | 42 | 44 | 100.0% | 4.8% |
| GSE33126 | 9 | 9 | 100.0% | 11.1% |
| GSE43841 | 6 |  |  | 100.0% |
| GSE53306 | 40 |  |  | 97.5% |
| GSE56789 | 40 |  |  | 90.0% |
| GSE68570 | 11 |  |  | 100.0% |
| **RNA_seq** | | | | |
| GSE50760 | 18 | 36 | 61.1% | 100.0% |
| GSE72819 | 73 |  |  | 100.0% |
| TCGA_coad† | 41 | 285 | 98.9% | 97.6% |

Note: *Empty cells indicate that there is no sample in the corresponding category;

^#^Empty cells indicate that there is no corresponding result because no the corresponding data;

^†^The colorectal adenocarcinoma samples from TCGA.
