# Supplementary figures and images for: Quantitative or qualitative transcriptional diagnostic signatures? A case study for colorectal cancer
Source: BMC Genomics. 2018 Jan 29;19:99. doi: 10.1186/s12864-018-4446-y (PMC5789529; doi:10.1186/s12864-018-4446-y)

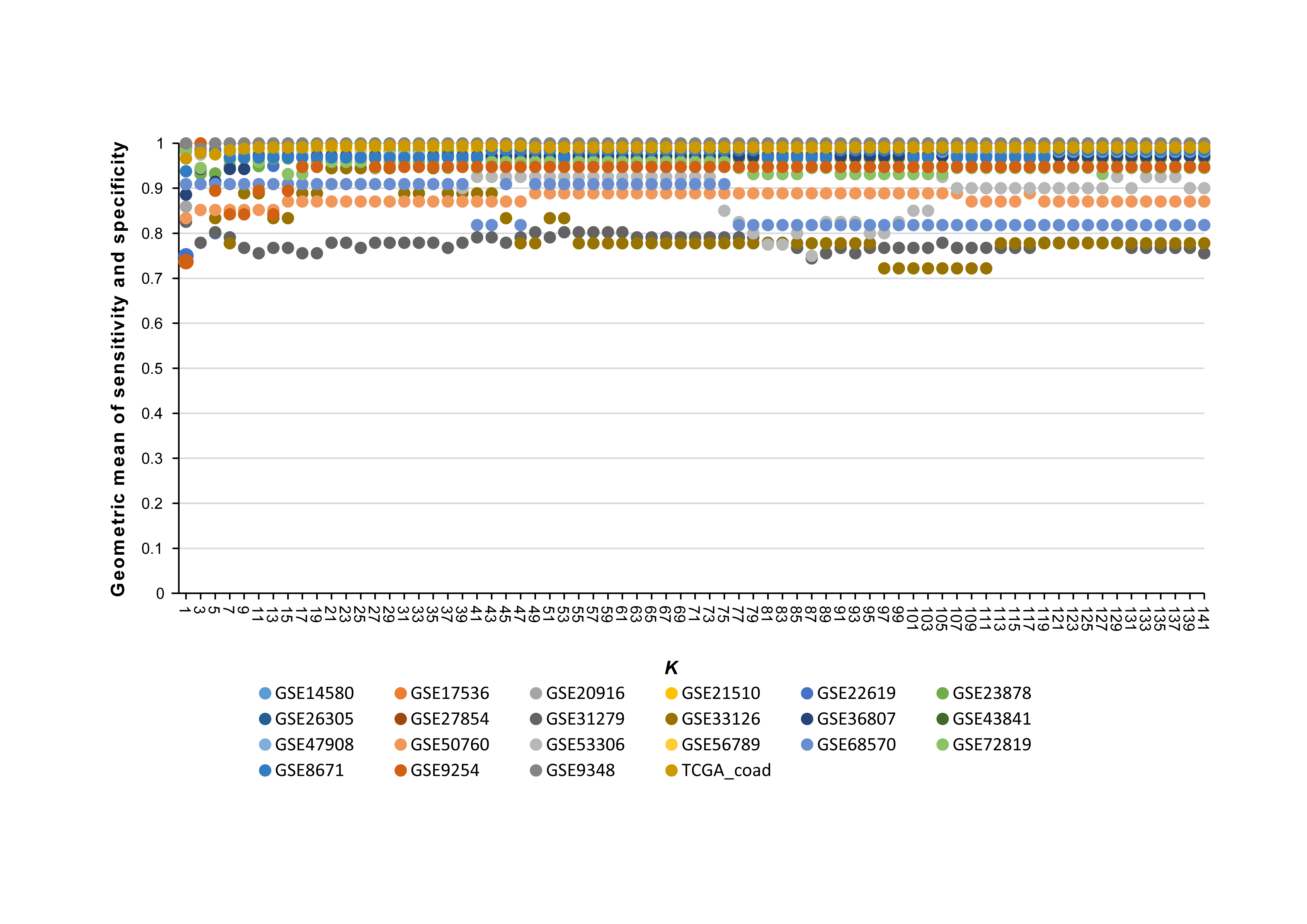

Supplement: Supplementary file 2 — Performances of all possible top-ranked k (from 1 to 141, k is an odd integer) gene pairs in the independent datasets measured by different platforms. The majority vote rule was used for classification. (TIFF 1848 kb) [file 12864_2018_4446_MOESM2_ESM.tif]
